# Care Perceptions in two ICU Nursing Care Delivery Models: A qualitative-comparative approach*

**DOI:** 10.17533/udea.iee.v40n3e15

**Published:** 2023-03-10

**Authors:** William Iván López Cárdenas, Esteban Gil Vidal, Rosa Milena Altamirano Ceron, Natalia Andrea Henao Murillo, Yuly Andrea Santa Mejía, Adriana Cristina Jurado Jiménez

**Affiliations:** 1 MSN, RN, Professor. Faculty of Nursing, Universidad de Antioquia, Medellín (Colombia) Corresponding author Email: wivan.lopez@udea.edu.co.. https://orcid.org/0000-0001-9369-562X Universidad de Antioquia Faculty of Nursing Universidad de Antioquia Medellín Colombia wivan.lopez@udea.edu.co; 2 MSN (c), RN. Faculty of Nursing, Universidad de Antioquia, Medellín (Colombia) E-mail: thisban.gilv@udea.edu.co https://orcid.org/0000-0001-9492-6426 Universidad de Antioquia Faculty of Nursing Universidad de Antioquia Medellín Colombia thisban.gilv@udea.edu.co; 3 BSN. Faculty of Nursing, Universidad de Antioquia, Medellín (Colombia) Email: ros.altamirano@udea.edu.co https://orcid.org/0000-0002-3981-0057 Universidad de Antioquia Faculty of Nursing Universidad de Antioquia Medellín Colombia ros.altamirano@udea.edu.co; 4 MSN, RN, Professor. Universidad Católica de Oriente, Rionegro (Colombia). Email: nathyh75@gmail.com https://orcid.org/0000-0001-5447-5616 Universidad Católica de Oriente Universidad Católica de Oriente Rionegro Colombia nathyh75@gmail.com; 5 MSN, RN, Hospital San Vicente Fundación, Medellín (Colombia) Director of Nursing Email: yuly.santa@sanvicentefundacion.com https://orcid.org/0000-0001-8647-1441 Hospital San Vicente Fundación Medellín Colombia yuly.santa@sanvicentefundacion.com; 6 MSN, RN, Hospital San Vicente Fundación, Medellín (Colombia) Director of Nursing Email: cristina.jurado@sanvicentefundacion.com https://orcid.org/0000-0003-2759-0189 Hospital San Vicente Fundación Medellín Colombia cristina.jurado@sanvicentefundacion.com

**Keywords:** critical care nursing, clinical nursing research, nursing team, nursing services, nursing care, health services administration, nursing care delivery model, enfermería de cuidados críticos, investigación en enfermería clínica, grupo de enfermería, servicios de enfermería, atención de enfermería, administración de los servicios de salud, modelo de prestación de cuidados de enfermería, enfermagem de cuidados críticos, pesquisa em enfermagem clínica, equipe de enfermagem, serviços de enfermagem, cuidados de enfermagem, administração de serviços de saúde, modelo de assistência de enfermagem

## Abstract

**Objective.:**

Analyzed in compared perspective perceptions about nursing care, nurse-patient interaction, and nursing care outcomes in two ICU nursing staff in a high-complexity hospital institution, whose Nursing are Delivery Models (NCDM) are differentiated by the proportion of nurses and nurse assistants (NA) per team and by the assigned tasks and responsibilities.

**Methods.:**

Particularist ethnography with adaptation to virtual methodologies. It included the sociodemographic characteristics of 19 nurses and 23 NA, 14 semi-structured interviews, review of patients’ clinical records, and a focus group. Coding, categorization, inductive analysis, validation of results with participants were conducted and thematic saturation was achieved.

**Results.:**

Four themes were identified: i) Professionalized care: a nursing of superior value; ii) senses and feelings of care; iii) nursing workload, generating factors andimpacts; and iv) nursing missed care as concrete expression of the nursing workload.

**Conclusion.:**

Compared nursing teams perceived nursing care in different ways, since it was experienced based on the assigned responsibilities and the possibilities of interaction with patients. Nursing care in the NCDM of the ICU with prevalence of direct bedside care by nurses with support from NA, it was perceived as holistic, comprehensive, and empathetic; whereas in the ICU with prevalence of delegated care to NA, it was related with administrative leadership and management of the ICU. Regarding the results, the NCDM of the ICU of direct bedside care by nurses showed better performance in patient safety and was closer to the skill level and legal responsibility of the nursing staff.

## Resumen

**Objetivo.:**

Analizar en perspectiva comparada las percepciones sobre el cuidado de enfermería, el relacionamiento con los pacientes y los resultados del cuidado en dos equipos de enfermería de UCI de una institución hospitalaria de alta complejidad, cuyos Modelos de Atención de Enfermería (MAE) se diferencian por la proporción de enfermeras y Auxiliares de Enfermería (AE) por equipo y por las tareas y responsabilidades asignadas.

**Métodos.:**

Etnografía particularista con adaptación a metodologías virtuales. Incluyó la caracterización sociodemográfica a 19 enfermeras y 23 AE, 14 entrevistas semiestructuradas, revisión de historias clínicas de pacientes y un grupo focal. Se realizó codificación, categorización, análisis inductivo, validación de resultados con participantes y se logró la saturación temática.

**Resultados.:**

Se identificaron cuatro temas: i) Cuidados profesionalizados: una enfermería de valor superior ii) sentidos y sentires del cuidado iii) carga de cuidado, factores generadores e impactos y iv) omisión del cuidado como expresión concreta de la carga de trabajo.

**Conclusión.:**

Los equipos de Enfermería comparados percibieron el cuidado de enfermería de maneras distintas, pues se vivenció a partir de las responsabilidades asignadas y las posibilidades de relacionamiento con los pacientes. En el MAE de la UCI donde predomina el cuidado directo de enfermeras con apoyo AE, se percibió el cuidado como atención holista, integral y empática, mientras que en la UCI donde predomina el cuidado delegado a AE se relacionó con el liderazgo administrativo y la gestión de la sala de UCI. En cuanto a resultados, el MAE de la UCI de cuidado directo por enfermeras mostró mejor desempeño en seguridad del paciente y fue más cercano al nivel de competencias y de responsabilidad legal de los equipos de enfermería.

## Resumo

**Objetivo.:**

Analisar em perspectiva comparativa as percepções sobre o cuidado de enfermagem, a relação com os pacientes e os resultados do cuidado em duas equipes de enfermagem de UTI de uma instituição hospitalar de alta complexidade, cujos Modelos de Assistência de Enfermagem (MAE) diferem pela proporção de enfermeiros e auxiliares de enfermagem (AE) por equipe e por tarefas e responsabilidades atribuídas.

**Métodos.:**

Etnografia particularista com adaptação a metodologias virtuais. Incluiu a caracterização sociodemográfica de 19 enfermeiros e 23 EA, 14 entrevistas semiestruturadas, revisão de prontuários e grupo focal. Codificação, categorização, análise indutiva, validação dos resultados com os participantes foram realizadas e a saturação temática foi alcançada.

**Resultados.:**

Foram identificados quatro temas: i) Cuidado profissionalizado: enfermagem de valor superior ii) sentidos e sentimentos do cuidado iii) sobrecarga do cuidado, fatores geradores e impactos e iv) omissão do cuidado como expressão concreta da carga de trabalho.

**Conclusão.:**

As equipes de Enfermagem compararam o cuidado de enfermagem percebido de diferentes maneiras, uma vez que foi vivenciado a partir das responsabilidades atribuídas e das possibilidades de relacionamento com os pacientes. No MAE da UTI onde predomina o cuidado direto por enfermeiros com apoio do EA, o cuidado foi percebido como cuidado holístico, integral e empático, enquanto na UTI onde predomina o cuidado delegado ao EA, estava relacionado à liderança administrativa e gestão da sala da UTI. Em termos de resultados, o MAE da UTI de cuidado direto por enfermeiros apresentou melhor desempenho na segurança do paciente e se aproximou mais do nível de competências e responsabilidade legal das equipes de enfermagem.

## Introduction

Within a global scenario characterized by nursing shortage, hospital institutions face the challenge of developing nursing care delivery models (NCDM) that involve diverse nursing staff qualification levels, guarantee high quality standards, safeness, cost effectiveness, and job satisfaction.(1) The NCDM are ways in which nursing practice can be organized to care for patients, principally in hospital settings.(2) 

In nursing literature, four classic NCDM are identified: Total Patient Care (TPC), Functional Nursing (FN), Team Nursing (TN), and Primary Nursing Care (PNC). (1) In the TPC model, the nurse is responsible for all patient care during a shift. (2) The FN model consists in the tasks distribution among the nursing staff in function of care complexity, knowledge, and skills required for its execution.(2) The TN model is comprised by personnel with different levels of experience and training, who also share the collective responsibility of care, optimizing skills, qualification, and team work.(3) In the PNC model, a nurse is responsible for coordinating a patient’s continuous care throughout the length of stay.(1) 

Characteristics of work environments, such as the proportion of nursing staff per unit, number of patients assigned, autonomy in decision making, and team work are predictive factors of results of the NCDM. Studies by Zhao *et al.,*(4) and Lake *et al.,*(5) showed inverse correlations between the characteristics of the work environments and omission of nursing care. Other studies have found associations among the composition of nursing staff, poor job satisfaction,(3) intention to switch jobs,(6) and the perception of low quality of care.(7) Teams conformed with a low proportion of nurses, large proportion of nurse assistants, and high nursing workload are associated with increased mortality,(8,9)length of stay,(8,9) readmissions,(9) increase of hospital-acquired infections, and adverse events.(8,10) In contrast, high assignment of nurses has been correlated with mortality reduction and positive perception of the quality of nursing care.(11)

Within the context of Colombian hospitals, the NCDM in Intensive Care Units (ICU) incorporate hybridizations of the aforementioned models. Regarding human resources, it must be highlighted that nursing work in Colombia is conducted under precarious working conditions, regional inequalities in the distribution of personnel, and lack of regulations on the functions of nurses -licensed personnel with university formation-, the functions of nursing assistants (NA) -unlicensed personnel with technical training of 2,600 hours- and on the patient- nursing ratio.(12) Regulatory gaps have had repercussions in displacing the work of nurses toward administrative-type actions and supervision of NA, which implies the forced allocation of most care provision to the NA and the conformation of work staff with a low proportion of nurses and a higher proportion of NA, affecting the quality and safety of care.(13) Moreover, in 2019, Colombia was ranked by the Organization for Economic Cooperation and Development as the country with the greatest shortage of nurses, with an indicator of 1.4 nurses per 1000 inhabitants.(14)

Some Colombian hospital institutions have been incorporating NCDM that involve a higher proportion of nurses for direct bedside care and a clearer definition between the roles and limits of the skills of nurses and nursing assistants. However, they have little scientific evidence to support their results, an aspect that motivated proposing this research, with potential contribution to the global discussion on professional practice environments that supports nursing autonomy and leadership, quality of care, and patient safety. The aim was to analyze in compared perspective, perceptions about nursing care, nurse-patient interaction, and nursing care outcomes in two ICU nursing staff in a high-complexity hospital institution, whose NCDM are differentiated by the proportion of nurses and nursing assistants per team and by the assigned tasks and responsibilities. 

## Methods

Type of study. Interpretative particularistic ethnographic based on Boyle(15) for the description and contextual interpretation of the meanings attributed by the nursing staff to care provided from two NCDM in ICU. Adaptations were made of the field work to the virtual context given the social distancing norms due to the COVID-19 pandemic. 

Setting. The research was conducted in two ICUs of a high-complexity Hospital located in Medellín-Colombia, which differ in the proportion of nurses, NA and in the functions assigned to each team. The study denominated as ICU-Nurse Team that with the highest proportion of nurses responsible for patient care and a lower proportion of NA dedicated to comfort functions; and as ICU-Assistants team that with a high proportion of NAs responsible for most of the patient care, and a low proportion of nurses with a large volume of administrative tasks, responsible for supervising NAs and some high-complexity nursing care.

Participants. The research team was made up by professors from Universidad de Antioquia, Universidad Católica de Oriente, Directors of Nursing from the Hospital, and nursing students. Participant inclusion criteria: nursing staff with at least one-year seniority in ICU-Nurses or ICU-Aides, performing bedside patient care functions or care management. 

Data collection. Data collection was carried out between June 2020 and May 2021. A virtual sociodemographic characterization survey was applied to 20 nurses and 33 NA, obtaining an 80% response rate. Thereafter, semi-structured interviews were conducted via Google Meet to five NA and nine nurses and a virtual focus group with six nurses and five NA. The observations were complemented with the revision of clinical records to identify differences with respect to functions, clinical records filled out, and completion of the care plan. 

Data analysis. By following Wolcott,(16) data coding and categorization was performed through inductive processes, bearing in mind the sampling and theoretical saturation. The findings were validated with the participants. 

Ethical aspects. The protocol was subjected to evaluation by the Ethics Committee of the university institution responsible for the research and obtained ethical endorsement in July 2019. Four virtual socialization meetings were held with the staff from both ICUs to introduce the study and the potential participants received an informed consent where they manifested their voluntary intention to participate. Also, identities were protected by substituting the names of the participants with alphanumeric codes.

## Results

**Figure 1:**
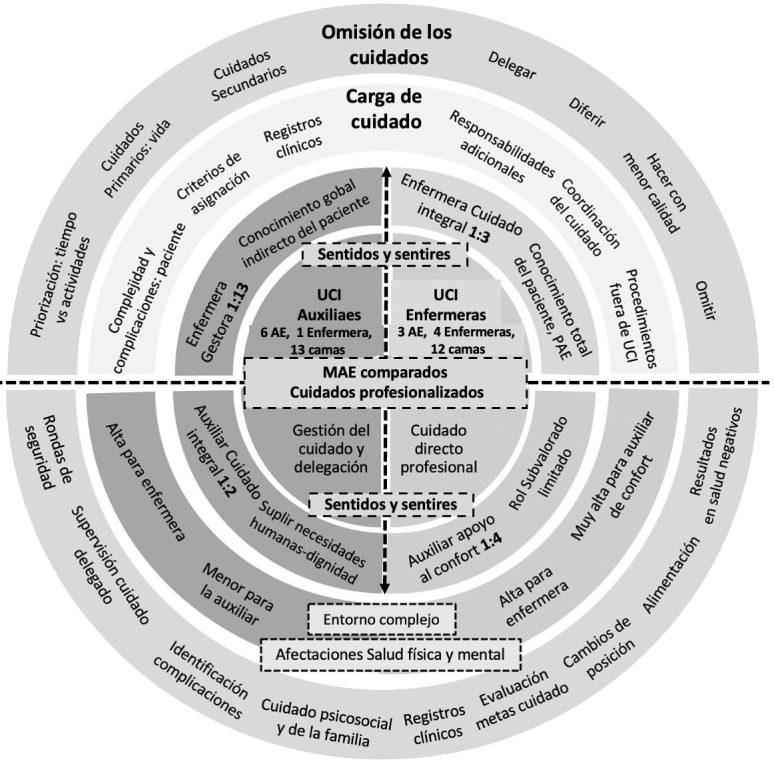
Schematic of thematic relations and analysis categories

Figure 1 presents the four emerging themes in the study stemming from a set of concentric circles from the center to the periphery. The central circle represents the theme of *Professionalized care: nursing of superior value*, which shows the differential characteristic of the NCDM and the teams compared. The following ring presents the *senses and feelings of care*, that is, the perception about care for nurses and NA in each of the teams according with the nursing staff: patient ratio and the functions assigned. The third and fourth rings present two categories related with the work environments where teams compared carry out their work: *nursing workload, generating factors and impacts*, and *nursing missed care as concrete expression of the work burden.*

Characteristics of the participants. Nurses constitute 64% of the staff in the ICU-Nurses and 25% in the ICU-Assistants. The proportion of NA is 36% in the ICU-Nurse team and 75% in the ICU-Assistants team. The mean time of job experience in both ICUs presents great variability, with values between 1 and 25 years for nurses in the ICU-Nurses team; between 1 and 6 years for nurses in the ICU- Assistants team; and between 1 and 25 years for NA in the ICU- Assistants team, indicating high personnel rotation. The NA in the ICU-Nurses team showed the lowest mean in work experience and age, with little variability in both variables, which suggests that majority in this group is quite young and has little work experience (Table 1).

**Table 1:** Sociodemographic characteristics of the study’s ICU staff

Variables		ICU-Nurses		ICU- Assistants	
		*N*	%	*N*	%
Job profile	Nurse	14	64	5	25
	NA	8	36	15	75
Sex	Masculine	2	9	1	5
	Feminine	20	91	19	95
Educational level	Technical-NA	8	36	15	75
	University	8	36	3	15
	Graduate	6	28	2	10
		Mean	SD*	Mean	SD*
Age	Nurse	34	7.7	31	4.5
	NA	23	2.3	32	7.7
Years of ICU experience	Nurse	3.5	4.2	2.8	2.2
	NA	2.6	0.8	8.0	7.5

* Standard deviation

### Theme 1. Professionalized care: superior value nursing

Between 2017 and 2020, a change in the performance of nursing work was carried out in the institution, framed in the provision of direct bedside care by nurses, to provide safe, integral, personalized and high-quality care. The NCDM implemented in the ICU-Nurses team sought to strengthen nurses’ skills for care and leadership and offer planned care, sustained on clinical judgment and disciplinary knowledge. From the organizational point of view, the intention was to position the hospital as a cutting-edge institution in care and patient safety: *one of the objectives of this form of bedside care work is to have reachable goals that are measurable in care for patients* (E001)*.* Prior to implementing the new NCDM, the hospital had a low proportion of nurses and the NAs were responsible for providing most of the care. The teams in the ICUs compared were comprised by one nurse and six NA for 12-13 beds (Table 2). The new NCDM was implemented gradually in half the hospital wards, diminishing the proportion of NA and increasing that of nurses 24 hours per day. The ICU-Nurses team established the 1:3 nurse-patient ratio and 1:4 NA-patient ratio and added a daytime coordinator, while the ICU-Assistants team is in the wards where the existing NCDM was not modified, with 1:13 nurse-patient ratio and 1:2 NA-patient ratio. 

**Table 2:** Comparison of the number of beds and nursing staff assigned in the ICUs compared

Service	N° beds	Professional profile	Staff assigned per shift		
			Before	During NCDM	Post-NCDM
ICU-Nurses	12	NA	6	3	6
		Nurse	1	4 + 1 daytime administrative coordinator	2 daytime and 1 at night
ICU-Assistants	13	NA	6	6	6
		Nurse	1	1	2 daytime and 1 at night

Implementation of the NCDM generated positive results for nurses and patients in the ICU-Nurses team. Nurses reported increased leadership, expertise, and technical skills to perform reflexive care practice. Care revived and reaffirmed their professional identities as care nurses: *we gain much, we gain leadership, experience, we manage to again revive that vocation for which we study, which is care for another* (E01)*.* The NCDM established care coordination mechanisms, like interprofessional rounds between physicians and nurses, safety rounds, measure packages, quality committees, actions for improvement and continuous training of the human talent. These measures permitted timely identification and intervention of warning signs and complications of patients. According with the participants, decreased rates of adverse events and infections associated with health care were evidenced in the ICU-Nurses team: *We have reflected this in the indicators. For example, we have had a year without pneumonia; we managed to have a year without bacteremia* (E02)*.*

During the implementation process of the NCDM by the ICU-Nurses team unplanned contingencies emerged as maladjustment experiences, fear, anxiety and resistance to change due to frequent modifications in processes, functions, work routines, and in the number of patients assigned: *We had a thousand meetings, we had a thousand accompaniments and that was very hard to organize. The first year was total chaos (B04).* The model’s learning and appropriation curves were slower than expected because the model was implemented with new personnel, without work experience, and because it required a cultural change of a historical role from administrative nurse to one of caring nurse: *It was a difficult process, given that most of the new arrivals were recent university graduates, without experience; then, the responsibility was on the nurses who had been previously in the Unit (C13).*

To end, it must be highlighted that the NCDM implemented in the hospital institution only has documentary support that describes the profile and functions of nurses and NA, hence, it does not have any referent of the body of nursing knowledge that guides their actions; although, in practice, tools are used, like the nursing-care plan. No measurements were made of the economic and epidemiological impact derived from improving the quality of care: *showing the savings that we have when we manage to lower infections, when we diminish adverse events, and when we diminish the length to stay of patients in the units is a path we still need to travel* (E03). 

### Theme 2. Senses and feelings of care

The number of patients and functions assigned configure differentiated ways of comprehending nursing care and nurse-patients interaction. With a 1:3 nurse-patient ratio, nurses in the ICU-Nurses team have a holistic vision of care based on the link and comprehensive knowledge of the patient and in the provision of planned care according to physical, social, and emotional needs, based on clinical judgment, nursing diagnoses, and care goals that guide interventions during the shift: *we use goals according to patient’s diagnosis and what I want to achieve in my shift with that patient… It is integral care and a very personalized approach* (E05). 

Among the nurses’ functions, there are the integral assessment of patients during each shift, elaboration and follow-up of the care plan in the clinical history, monitoring, identification and intervention of risks and complications, interaction and education of the family, accompanying diagnostic procedures, administration of medications, care of the air way, invasive procedures, such as insertion of probes, catheters, dressings and assistance in Cardiopulmonary Resuscitation. The ICU-nurses team has a coordinator on business days and daytime hours responsible for the ward’s administrative management, assignment of patients to work teams, coordination of care with the medical staff, and evaluation of the results of care. The coordination role favors nurses having more time available to dedicate to caring for patients.

The NAs from the ICU-Nurses team have a 1:4 NA-patient ratio, complement nursing care with activities of comfort, monitoring, and basic patient care on aspects like hygiene, feeding, prevention of pressure wounds, balance of liquids, and hourly control of vital signs. The NA consider the ICU a complex space that is motivating and generator of learning. The technological setting and the patient’s complexity are perceived as challenges to develop skills and knowledge, which instills fear during the initial approaches to the ICU, considering that the vast majority has little prior work experience: *At the beginning you get scared. It is a bit hard because of the types of patients we get* (A01). With respect to their work, the NA from the ICU-Nurses team manifested the sense of under-estimation of their capacity to provide care when compared with the NA from the ICU-Assistants team, given that the first receive lower salaries and their role focused on comfort is contrary to their job expectations in a technological setting, like the ICU.

In contrast, in the ICU-Assistants team, one nurse is responsible for 13 patients and experiences the practice from a conception of administrative leadership, given that most of the nurse’s time is spent on clinical management functions, although performing some specialized nursing procedures, like taking blood cultures, blood gases, complex cures, and assistance in invasive medical procedures. This form of care is characterized by the obligatory delegating a broad range of care interventions to the NA without bearing in mind their levels of knowledge and skills, by supervising the work of the NA, and by a superficial level of the nurse’s involvement in knowing the patient and in planning the care; in many shifts, they do not even get to know their patients in charge: *the current model that I am in is more of leadership and management, although you get to accompany many procedures, it has a more global vision of the patients*… *it is difficult to care for all patients in integral manner* (E04). 

Finally, the ICU-Assistants team showed a 1:2 NA-patient ratio; the NA must assume providing most of the nursing care required by their patients that include activities not in accordance with their level of knowledge and skills, like administration of medications, sedation, inotropes and parenteral nutrition, aspiration of secretions, early identification of complications, manipulation of invasive devices and dressings. Constant bedside accompaniment to patients allows them to conceive care as the possibility of accompaniment and satisfaction of needs from human dignity: *What does nurse care mean? For me, I believe it is everything in those patients because we fulfill all the needs or human worthy conditions, like hygiene, being comfortable, that someone is looking out for you, being here with you, feeling you, talking with you* (A02).

### Theme 3. Nursing workload: generating factors and impacts

Nursing workload was a common characteristic in the ICUs compared. It derives from factors like assignment criteria, number and severity of patients, parallel responsibilities to care, and from the possibility of effective coordination of the work teams. The allocation of patients is made by geographical proximity in both ICUs, without using criteria, like measurement of workload or severity of the patients, which leads to unequal distribution of the most-complex patients among the staff in the ICUs. For the nurses in the ICU-Assistants team, the workload-generating activities in which they spend most of their work time are taking blood cultures, accompanying the medical rounds of 13 patients, and assisting physicians during invasive procedures or during cardiopulmonary resuscitation. Nurses in the ICU-Nurses team consider their caregiving responsibilities to be overwhelming given that compared with the NA in the ICU-Assistants team, they are responsible for more patients and bedside-care functions, like elaborating the care plan: *since we started the model, one tries to do things more consciously and tries to do them better, it takes a little longer (E02).*

The NA in the ICU-Nurses team consider the assignment of four patients to surpass their capacity to provide the care required, thus, they experience physical and emotional fatigue, stating that they use up half the shift in bathing their patients. The NA in the ICU-Assistants team reported feeling comfortable and without overburden to provide care to their two patients assigned, except for the person who has an additional patient, which produces mental overburden and affectation in the quality of care provided. *There are 13 patients, they day you had that odd-patient there was a more mental workload, it is not the same to take care of two than to take care of three. The quality of care is not the same* (A04)*.*

The NA and nurses in both ICUs have additional responsibilities that increase the workload like cleaning equipment, counting medications and medical supplies, custody of elements of the service, accompanying the transfer of patients to surgeries or diagnostic exams, and elaborating multiple registries in the clinical chart. The nursing staff arrives earlier than usual to their work day or extend their work day until after the work shift is over to comply with these obligations. *Many colleagues ended up leaving the institution two hours later because they had many things to do, especially the system’s registry* (E04)*.*

Coordination of care among the nursing staff is a determining factor of the increase or decrease of the workload. When the NA in the ICU-Assistants team have to transfer any of their patients to a procedure outside the ICU, they get support from their colleagues to care for the other patient. In the ICU-Nurses team, it was expected for NAs and nurses to work in pairs to provide care, like changes in position, however, this articulation is not achieved because both profiles are attending to the urgent needs of their patients and because all NA having four patients assigned, must coordinate their work with two nurses. *I have three patients with one nurse and the other with a different nurse. So, it was two nurses that called out “come” and that became total chaos* (A03). 

Both ICUs are perceived as environments that generate emotional and physical burden due to the constant closeness with the deaths of patients, restriction of their own needs like going to the restroom or eating to prioritize those of the patient, besides the physical fatigue that prevents enjoying other moments beyond work: *one always prioritizes the patients first prior to going to the restroom, before going for a meal; when you least expect, it is four in the afternoon and you have not had lunch* (E06). 

### Theme 4. Missed nursing care as concrete expression of the work burden

This understood as the impossibility to provide all the care patients require within a context of overload, which obligates the nursing staff from both ICUs to make decisions regarding the care that must be prioritized, having to delay or miss the rest of the interventions. As a common point, the nursing staff prioritize patients to care for and care to be provided based on criteria, like the state of health and complications of the patients. Care for unstable patients, taking blood cultures, and administration of medications are fundamental to sustain life and cannot be missed. In contrast, stable patients receive less time of care and, consequently, lower quality of care: *you stay in that cubicle all day and the other patients lose out… you cannot take care of the other patient* (A03).

On a second level of prioritizing, there is a group of cares denominated by the participants as secondary, covering psychosocial and family care; activities of comfort, clinical records (including care plans), and actions for patient surveillance. These actions tend to be omitted, delayed, diminishing their frequency, or carried out with less quality: *Primary care would be the fundamental care, those that must be done now or now! That the patient has low blood pressure, it is now or now! I must stabilize the blood pressure, administer an inotrope... But the others that I am leaving would be like secondary care... the psychosocial issues of the person, which integrate the patient’s family* (E5). Lack of time do not allow nurses in the ICU- Assistants team supervising and accompanying the NAs, conducting patient safety rounds, and filling out the care plan (an aspect verified in the revision of medical records). The aspects mentioned have negative implications for the quality and coordination of care between the nurse and her NAs: *we assign specific supervision functions, conduct safety rounds, evaluate the BUNDLES; we do not even comply with those functions because time absorbs us so much... there are functions assigned to the NA by the hospital… and one notices that there is no time for supervision* (E04).

In turn, the NA in the ICU- Assistants team prioritize care of the biological sphere, like administration of medications, aspiration, and sedation. Care most-often delayed or omitted include feeding, changes of position, and registries in the system and these delays occur when they have to leave the ward to accompany another patient to a procedure or when another patient shows some complication. Awake patients are considered as demanding care, such as feeding: *patients that are quite autonomous we have to help them to feed very often and it is very complicated because the feeding of these patients gets delayed while one is doing something else (B24)*.

In the ICU-Nurses team, missing care is expressed by nurses not being able to comply with the goals proposed in the care plan: *If I don't have the time to dedicate the 15 minutes he needs for the breathing incentive, I won't achieve the goal, which was to remove his oxygen (E05)*. The seriousness of a patient and the follow-up to diagnostic procedures outside the ward are the factors that most generate workload and missed care.

Coordination and continuity of care are generating factors of undone activities for nurses in the ICU-Nurses team. Revision of clinical histories is a frequently delayed activity, and in some cases, when physicians do not communicate their prescription to nurses, these become aware of changes in the management of patients at the end of the shift. With respect to the continuity of care, nurses are assigned different patients during each shift and rarely do they have a chance to give continuity to their goals and interventions. There is also no continuity for the care plan among nurses in the different shifts, given that each establishes their own care goals and actions without contemplating those established by their colleagues from the previous shift. In turn, NAs in the ICU-Nurses team reported that care most-often delayed, omitted, or performed with lower quality include feeding, hygiene, changes of position, measurement and disposal of collection systems: *you don’t have enough time to spend every hour removing residues from the bladder catheter and, for example, we must perform the change of position every two hours, not lately* (B001). In patients, omission is expressed in the times they stop receiving care, with consequences, like occurrence of adverse events, prolonged length of stay, and complications: *we had completed one year without pneumonia, we reviewed the case and effectively the hygiene [oral cavity] of these patients had not been done on certain moments (E02).*

To conclude, providing care against the clock generates feelings of guilt and frustration in the nursing staff from both ICUs, given that missed care distances them from desired ideal of care: *It is too frustrating, as much as I would like to split myself in three and do to everyone what needs to be done, sometimes things have to be done quickly, not with the love and dedication one would want do it* (A03). 

### Termination of the NCDM in the hospital

In 2020, the gradual dismantling of the NCDM began for reasons of institutional financial sustainability. ,At the end of the NCDM the previous NAs standard and their integral patient care responsibilities were re-established . It also returned to the assignment of nurses prior to the model, although for the ICU-Nurses team and ICU-Assistants team a nurse was added as daytime support (Table 2). The staff from the ICU-Nurses team expressed missing the care organization promoted during NCDM validity, given that it allowed nurses to get deeply involved in care, as they learned at the university, while the NAs returned to a higher level of responsibility with patients under a scheme of supervision, which is why they consider not having the accompaniment of nurses for cooperative care. The nurses from the ICU-Assistants team manifested that, in spite of having support from an additional nurse since the re-structuring of the NCDM, the possibility of performing integral care similar to that carried out in the ICU-Nurses team continues being quite limited due to the number of patients assigned.

## Discussion

Comparing the ways of working from the four categories exposed permits identifying two central points of debate: implementation of an NCDM close to the professional practice desired and the influence of work environments on the quality and safety of care. The NCDM implemented in the ICU-Nurses team configured a role of care nurses by permitting a distribution of functions according with the level of legal responsibility, knowledge, and competence among the profiles integrating the work staff. Responsibility for providing care in this model was assigned to nurses, an aspect that reaffirmed their identity as caregivers, enhanced their autonomy and leadership, allowing them to offer empathetic and planned care from a holistic perspective. The NAs participated within a model with a role of articulation and support to nurses in actions of basic monitoring, hygiene, and comfort, which constitute responsibilities according with their level of training and skills. 

According with Fawcett,(2) this NCDM shares some characteristics of the Team Nursing upon articulating nurses and NAs in individualized and integral patient care, optimizing the skills and resources of the work team for the quality of care. The perceptions about the care provided by the nurses in the ICU-Nurses model coincide with the study by Zamaniniya *et al.,*(17) who state that the practice of humanistic care in ICU generates feelings, such as personal growth, self-realization, satisfaction, motivation and development of ethical competencies in nurses. In this sense, authors, like Stavropoulou *et al.,*(18) identified as pillars of empathic care in the ICU the capacity to listen to patients, experience their feelings, understand them and help them to ease uncertainty.

The NCDM by the ICU-Assistants team assigned the nurse a role as manager and ward supervisor, which implies superficial knowledge of patients and of their care needs due to the forced delegation of the majority of care to the NAs, whose functions and responsibilities assigned overcome their knowledge and level of competence. These particularities fit within the model of Functional Nursing described by Fawcett,(2) where care is fragmented among staff members and is distanced from holistic care. The defining characteristics of this NCDM were identified in the analysis of the nursing panorama in Colombia as conducted by Ortega and Jiménez,(13) who found that most of the care in ICU requiring specialized knowledge and techniques are performed by NA; likewise, the authors indicate that nurses must delegate to NAs approximately 44% of the care of the physiological sphere that are considered by themselves as non-delegable. They also stated that the administrative work in which nurses invest the greatest amount of time could be carried out by health administrative assistants. This global problematic also emerged during the systematic revision by Blay and Roche,(19) who identified more than 200 activities delegated to NA that go beyond their training level and are conducted under limited supervision by nurses.

The comparison made permits concluding that the NCDM by the ICU-Nurses team approaches the professional practice desired in the Colombian context, understanding that the organization of work between nurses and NAs was coherent with their competencies and skills, offering better standards of quality and safety in care. However, it is worth highlighting some elements that limited the potential of the NCDM as organization tool of nursing work: a volume of patients assigned to nurses and NAs, which generates workload and omissions or delays in care, the absence of a guiding framework of the NCDM sustained in nursing models and theories, and the lack of measurements related with their economic and epidemiological outcomes.

As second point of debate, a common feature for both NCDM was work environments that compromised care quality and safety. In both ICUs, the number of patients and responsibilities assigned (some not related directly with care) generated multiple demands, limited the time and resources available from the nursing staff to provide holistic care, generated fatigue and overwhelm, and could be related with the personnel rotation identified in the description of the participants’ characteristics. These factors have been identified in the analysis of barriers to empathic care by Stavropoulou *et al.*,(18) among which there are lack of staff, increased workload, and fatigue. Some feelings emerging from the reports regarding the work overload agree with the findings by Chetty(20) and Banda *et al.*,(21) who indicate that the workload causes a negative psychological impact on ICU nurses, manifested by anger and discouragement, emotional overburden, stress and impossibility to conciliate personal and work life. Chetty(20) also referred to the intention of leaving work and to the staff rotation as outcomes of the overburden experienced by the nurses. 

Additionally, the study by Subhi(22) highlights that the nursing staff must be in charge of many things at the same time, making it difficult to complete all their activities, which affects the capacity to care for others. Thus, the omission or delay of nursing care is introduced as a specific indicator of work overload, which affects negatively on the quality of care and patient safety. As with the system of prioritizing care identified by Banda as response to workload,(21) the ICUs compared privileged care of the biological sphere over the patient’s psychosocial needs, which could result in a less humanistic and more instrumental vision of care. In the nurses from the ICU-Nurses team omissions in the follow-up of care plans or education of the family distanced the work of nurses from holistic care, while for nurses from the ICU-Aides team, the impossibility to plan care, supervise that delegated, and implement prevention actions, like safety rounds or follow-up to bundles had negative implications on patient safety. For the NAs from both ICUs, omission of care related with hygiene and comfort resulted in adverse events, such as pressure wounds and pneumonia associated with ventilation. The members from both nursing teams reported feelings of frustration due to omission and delay of care, which distanced them from their ideal of patient care. 

Similar findings have been reported in other qualitative research, evidencing that missed care is a global problematic and a challenge to improve the quality of health care. In this sense, Suhonen *et al.,*(23) describe prioritizing of medical needs as an aspect that removes the nursing staff from a humanistic practice. Lake *et al.,* (5) and Kalish(24) reported that care that is delayed or omitted most frequently encompass activities of daily life, like hygiene, changes of position and comfort, delay in some medications, communication and emotional support, education to the patient and the family. Feelings of guilt and job dissatisfaction derived from the missed care, defined by Janatolmakan and Khatony(25) also coincide with the feelings expressed by nurses and NA participating in the study.

To close this second point of debate, we summarize the findings about generating factors of work overloud and omission of care that emerged within the context of the NCDM compared and which have been identified in other qualitative research: characteristics of the patient (complexity, emergency situations),(21) of the nurse (time of experience, skills in prioritizing and delegating, articulation with NAs in decision making about care)(21) and of the work environment (staff shortage, workload, time invested in tasks not related with nursing, coordination of care with NA and medical staff).(25)

To end, the following were indicated as study limitations: i) no institutional information was found that accounted for the planning and operation of the NCDM proposal of the ICU-Nurses team to triangulate such with the reports by the nursing staff and ii) adaptation of the field work to virtual methodologies in response to the COVID 19 pandemic restricted the possibility conducting participant observation and face-to-face interviews, aspects that would have permitted a more-profound vision of the institutional context, work environments, and the implementation of the NCDM compared.

Regarding the contributions of the research, the results invite to improving hospital care processes by implementing NCDM and work environments that promote nursing autonomy and leadership to reach high levels of care quality and patient safety. These results also raise new questions to contribute from the investigation to the knowledge of the ethical and moral implications of missed care, the experience patient-centered care, and to deepen from quantitative methods the relationship among the NCDM, work environments, assignment of staff, patient and nursing staff outcomes. Consequently, it becomes necessary to derive knowledge from the disciplinary research to assist regulations and make administrative decisions on nursing work in the local and global scenarios.

In conclusion, the nursing teams compared perceived nursing care differently, given that this is experienced from the responsibilities assigned and possibilities of relating with patients. In the NCDM of the ICU-Nurses team, care was perceived as holistic, comprehensive, and empathetic care; while in the ICU-Assistants team, care was related with administrative leadership and management of the ICU ward. Regarding results, the NCDM in the ICU-Nurses team showed better performance in patient safety and was closer to the level of skills and legal responsibility of the NAs and nurses, in comparison to the NCDM in the ICU-Assistants team.

The experience of the NCDM developed in the ICU-Nurses team provides elements to rethink new possibilities of organizing nursing work in Colombian hospitals that are coherent with the level of training, experience, and skills of the teams of nurses and NAs, with the number of patients and functions assigned, which promote holistic care and reaffirm the autonomy, leadership, and caregiver identity of the nurses. Considering the positive results for the nursing staff and for patients of the NCDM of ICU-Nurses teams, it is recommended that the hospital institution, where said experience was carried out, to conduct complementary research from a quantitative approach and in retrospective perspective that permits evidencing the economic and epidemiological impact of enhancing the quality of nursing care, as well as establishing the impact of bedside care by nurses in the patient’s health results.

## References

[1] Prentice D, Moore J, Desai Y (2021). Nursing care delivery models and outcomes: A literature review. Nurs. Forum.

[2] Fawcett J (2021). Thoughts About Models of Nursing Practice Delivery. Nurs. Sci. Q.

[3] Goh PQL, Ser TF, Cooper S, Cheng LJ, Liaw SY (2020). Nursing teamwork in general ward settings: A mixed-methods exploratory study among enrolled and registered nurses. J. Clin. Nurs.

[4] Zhao Y, Ma D, Wan Z, Sun D, Li H, Sun J (2020). Associations between work environment and implicit rationing of nursing care: A systematic review. J. Nurs. Manag.

[5] Lake ET, French R, O'Rourke K, Sanders J, Srinivas SK (2020). Linking the work environment to missed nursing care in labour and delivery. J. Nurs. Manag.

[6] Cho SH, Lee JY, You SJ, Song KJ, Hong KJ (2020). Nurse staffing, nurses prioritization, missed care, quality of nursing care, and nurse outcomes. Int. J. Nurs. Pract.

[7] Bridges J, Griffiths P, Oliver E, Pickering RM (2019). Hospital nurse staffing and staff-patient interactions: an observational study. BMJ Qual. Saf.

[8] Almenyan AA, Albuduh A, Al-Abbas F (2021). Effect of Nursing Workload in Intensive Care Units. Cureus.

[9] Lasater KB, Sloane DM, McHugh MD, Cimiotti JP, Riman KA, Martin B, Alexander M, Aiken LH (2021). Evaluation of hospital nurse-to-patient staffing ratios and sepsis bundles on patient outcomes. Am. J. Infect. Control.

[10] Duffield C, Twigg D, Roche M, Williams A, Wise S (2019). Uncovering the Disconnect Between Nursing Workforce Policy Intentions, Implementation, and Outcomes: Lessons Learned from the Addition of a Nursing Assistant Role. Policy Polit. Nurs. Pract.

[11] Aiken LH, Sloane D, Griffiths P, Rafferty AM, Bruyneel L, McHugh M, Maier CB, Moreno-Casbas T, Ball JE, Ausserhofer D, Sermeus W, RN4CAST Consortium (2017). Nursing skill mix in European hospitals: cross-sectional study of the association with mortality, patient ratings, and quality of care. BMJ Qual. Saf.

[12] Colombia, Ministerio de Salud y Protección Social (2022). Resolución 0755 de 2022, por la cual se adoptan la Política Nacional de Talento Humano de Enfermería y el Plan Estratégico 2022-2031" para el fortalecimiento del talento humano en salud.

[13] Ortega M, Jiménez A (2020). Situación actual de enfermería en Colombia una reflexión. Rev. Ocupación Humana.

[14] OECD (2021). Health at a Glance 2021: OECD Indicators.

[15] Boyle J, Morse J. (2003). Asuntos críticos en los métodos de investigación cualitativa.

[16] Wolcott H (1994). Transforming Qualitative Data: Description, Analysis and Interpretation.

[17] Zamaniniya Z, Khademi M, Toulabi T, Zarea K (2021). The outcomes of humanistic nursing for critical care nurses: A qualitative study. Nurs. Midwifery Stud.

[18] Stavropoulou A, Rovithis M, Sigala E, Pantou S, Koukouli S (2020). Greek nurses' perceptions on empathy and empathic care in the Intensive Care Unit. Intensive Crit. Care Nurs.

[19] Blay N, Roche MA (2020). A systematic review of activities undertaken by the unregulated Nursing Assistant. J. Adv. Nurs.

[20] Chetty K (2021). The Cost of Caring: ICU Workload Stressors and the Saudi Arabian Nurse. Health Sci. J.

[21] Banda Z, Simbota M, Mula C (2022). Nurses' perceptions on the effects of high nursing workload on patient care in an intensive care unit of a referral hospital in Malawi: a qualitative study. BMC Nurs.

[22] Subhi H (2022). Enablers and challenges of caring in the Intensive Care Unit-Part 2: In relation to nurses. J. Nurs. Educ. Pract.

[23] Suhonen R, Stolt M, Habermann M, Hjaltadottir I, Vryonides S, Tonnessen S (2018). Ethical elements in priority setting in nursing care: A scoping review. Int. J. Nurs. Stud.

[24] Kalisch BJ (2006). Missed nursing care: a qualitative study. J. Nurs. Care Qual.

[25] Janatolmakan M, Khatony A (2022). Explaining the experience of nurses on missed nursing care: A qualitative descriptive study in Iran. Appl. Nurs. Res.

[26] Tang CJ, Lin YP, Chan EY (2021). 'From Expert to Novice', Perceptions of General Ward Nurses on Deployment to Outbreak Intensive Care Units during the COVID-19 Pandemic: A Qualitative Descriptive Study. J. Clin. Nurs.

